# High neutrophil-to-lymphocyte ratio associated with progression to critical illness in older patients with COVID-19: a multicenter retrospective study

**DOI:** 10.18632/aging.103582

**Published:** 2020-07-30

**Authors:** Jiangshan Lian, Ciliang jin, Shaorui Hao, Xiaoli Zhang, Meifang Yang, Xi Jin, Yingfeng Lu, Jianhua Hu, Shanyan Zhang, Lin Zheng, Hongyu Jia, Huan Cai, Yimin Zhang, Guodong Yu, Xiaoyan Wang, Jueqing Gu, Chanyuan Ye, Xiaopeng Yu, Jianguo Gao, Yida Yang, Jifang Sheng

**Affiliations:** 1 State Key Laboratory for Diagnosis and Treatment of Infectious Diseases, National Clinical Research Center for Infectious Diseases, Collaborative Innovation Center for Diagnosis and Treatment of Infectious Diseases, Department of Infectious Diseases, The First Affiliated Hospital, College of Medicine, Zhejiang University, Hangzhou, China; 2 Department of Gastroenterology, The First Affiliated Hospital, College of Medicine, Zhejiang University, Hangzhou, China

**Keywords:** SARS-CoV-2, COVID-19, older patients, neutrophil-to-lymphocyte ratio, risk factor

## Abstract

This retrospective cohort study aimed to investigate the correlation of the neutrophil-to-lymphocyte ratio (NLR) with critical illness in older patients with COVID-19, and evaluate the prognostic power of the NLR at admission. We enrolled 232 patients with COVID-19, aged ≥60 y, in Zhejiang province from January 17 to March 3, 2020. Primary outcomes were evaluated until April 13. Cox regression was performed for prognostic factors. Twenty-nine (12.5%) patients progressed to critical illness. Age, shortness of breath, comorbidities including hypertension, heart disease, and chronic obstructive pulmonary disease, higher NLR, lower albumin levels, and multiple mottling and ground-glass opacity were associated with progression. In the multivariate analysis, older age (hazard ratio [HR] 1.121, confidence interval [CI] 1.070-1.174, P<0.001), heart disease (HR 2.587, CI 1.156-5.787, P=0.021), higher NLR (HR 1.136, CI 1.094-1.180, P < 0.001), and multiple mottling and ground-glass opacity (HR 4.518, CI 1.906-10.712, P<0.001) remained critical illness predictors. The NLR was independently associated with progression to critical illness; the relationship was significant and graded (HR: 1.16 per unit; 95% CI: 1.10-1.22; P for trend < 0.001). Therefore, NLR can be adopted as a prognostic tool to assist healthcare providers predict the clinical outcomes of older patients suffering from COVID-19.

## INTRODUCTION

In December 2019, a novel coronavirus called severe acute respiratory syndrome coronavirus 2 (SARS-CoV-2) was first identified in Wuhan, China [1–3]. Infection with the virus leads to coronavirus disease (COVID-19), which is characterized by rapid human-to-human transmission and varied degrees of fatality, due to acute respiratory distress syndrome, multi-organ failure, and other serious complications [4, 5]. The global spread of this pandemic has been rapid since March 2020. As of mid-April 2020, more than 2 million individuals had been diagnosed with the disease, leading to over 150,000 deaths.

In our previous study, we found that older patients with COVID-19 had significantly greater disease severities, as well as higher rates of critical-type disease and intensive care unit (ICU) admission than their younger counterparts outside Wuhan [6]. Wang et al. [7] found that patients treated in the ICU were older than those without ICU treatment in Wuhan. In the United States, Garg et al. [8] demonstrated that older adults had elevated rates of COVID-19-associated hospitalization, and the majority of people hospitalized with COVID-19 had underlying medical conditions. In Italy, a majority of critically ill patients with laboratory-confirmed COVID-19 who were admitted to ICUs were older men, and a large proportion of them required mechanical ventilation and high levels of positive end-expiratory pressure; the associated ICU-related mortality was 26% [9].

Many studies have shown that older age is an independent risk factor for fatal outcomes in patients with COVID-19 [10–12]. Wang et al. investigated the characteristics of elderly patients with COVID-19 and the associated prognostic factors, and found that the presence of acute respiratory distress syndrome was a strong predictor of death. In addition, high lymphocyte levels were predictive of better outcomes [13]. Lymphopenia is a risk factor for severe illness and death among patients with COVID-19 [14].

The neutrophil-to-lymphocyte ratio (NLR) can be easily determined from the full blood count, and has been reported to be closely related to patients’ overall inflammatory status.

Increasing NLR values are risk factors of mortality in not only infectious disease settings but also cancer [15, 16]. A study showed that the NLR is an independent risk factor of mortality in hospitalized patients with COVID-19 [17]. The identification of a good indicator of disease progression can aid clinicians in improving the effect of therapy and reducing the mortality related to COVID-19 without excessive medical resource use. Whether the NLR can predict progression to critical illness in older patients with COVID-19 requires further elucidated.

In this study, we investigated the correlation of the NLR with critical illness in older patients with COVID-19, to evaluate the prognostic power of the NLR at admission in the prediction of progression to critical illness.

## RESULTS

### Demographic and epidemiologic characteristics

In this study, 232 older (≥60 years) patients with confirmed COVID-19 were enrolled from January 17, 2020 to March 3, 2020 in Zhejiang province. Patients’ clinical outcomes were followed-up until April 13, 2020. As shown in Table 1, the median ages in the mild, severe, and critical disease groups were 66 years (interquartile range [IQR]: 63-70), 66 years (IQR: 62-71) and 72 years (IQR: 68-81). The critical group showed a significantly higher age than the mild and severe groups (*P*<0.001). The proportions of hypertension and heart disease in the critical group were 72.41% and 55.17%, respectively, which were significantly higher than those noted in the mild and severe groups (*P*<0.001). One case (0.71%) with mild disease, two (3.14%) with severe disease, and six (20.69%) with critical disease had chronic obstructive pulmonary disease (COPD) (*P*<0.001). There were no significant differences in the other coexisting medical conditions across the three groups, including the rates of diabetes, asthma, cancer, chronic liver disease, chronic renal disease, and immunosuppression.

**Table 1:** Demographic, epidemiologic, and clinical characteristics of the different subtypes in older patients with COVID-19.

**Characteristic**	**Mild type (n=140)**	**Severe type (n=63)**	**Critical type (n=29)**	***P* value**
**Age** (years)	66(63-70)	66(62-71)	72(68-81)	<0.001
Distribution				
60-70 y	102(72.86)	45(71.435)	7(24.14)	<0.001
70-80 y	30(21.43)	14(22.22)	13(44.83)	0.025
≥80 y	8(5.71)	4(6.35)	9(31.03)	<0.001
**Sex (male)**	62(44.29)	28(44.44)	19(65.52)	0.102
**Body mass index** (kg/m^2^)	23.52(21.23-25.39)	24.34(22.25-25.16)	24.51(22.89-26.62)	0.227
**Current smoker**	17(12.14)	4(6.35)	4(13.79)	0.418
**Exposure history in Wuhan**	25(17.86)	18(28.57)	5(17.24)	0.194
**Contact with patients**	82(57.14)	25(39.68)	12(41.37)	0.023
**Family cluster**	50(35.71)	20(31.75)	10(34.48)	0.859
**Time from illness onset to first hospital admission** (days)	3(1-6)	5(2-7)	3(1-5)	0.048
**Coexisting disorder**				
Any	76(54.29)	25(38.68)	13(44.83)	0.132
Hypertension	57(40.71)	22(34.92)	21(72.41)	0.004
Heart disease	8(5.71)	7(11.11)	16(55.17)	<0.001
Diabetes	29(20.71)	9(14.29)	4(13.79)	0.431
asthma	1(0.71)	1(1.59)	2(6.90)	0.076
Chronic obstructive pulmonary disease	1(0.71)	2(3.14)	6(20.69)	<0.001
Cancer	2(14.29)	1(1.59)	1(3.45)	0.766
Chronic liver disease	4(2.86)	4(6.35)	2(6.90)	0.397
Chronic renal disease	3(2.14)	1(1.59)	2(6.90)	0.313
Immunosuppression	0(0)	2(3.17)	0(0)	0.064
**Symptoms on admission**				
Fever	110(78.57)	55(87.30)	25(86.21)	0.105
Cough	94(67.14)	38(60.2)	22(75.87)	0.461
Sputum production	46(32.86)	26(41.27)	15(51.72)	0.148
Hemoptysis	2(1.43)	1(1.59)	1(3.45)	0.766
Sore throat	13(9.29)	8(12.70)	2(6.90)	0.598
Nasal obstruction	2(1.43)	0(0%)	1(3.45)	0.404
Myalgia	12(8.57)	8(12.70)	4(13.79)	0.552
Fatigue	19(13.57)	11(17.46)	8(27.59)	0.202
Gastrointestinal symptoms	12(8.57)	6(9.52)	7(24.14)	0.06
Headache	5(3.57)	6(9.52)	0(0%)	0.073
Shortness of breath	1(0.71)	7(11.11)	12(41.38)	<0.001

Data are presented as medians (interquartile ranges), n (%) and n/N (%).

### Clinical features and laboratory abnormalities

On admission, the majority of cases showed decreased or normal leucocyte levels in all subtypes, as shown in Table 2. The median neutrophil levels in the mild, severe, and critical groups were 3.22×10^9^ /L [IQR: (2.59-4.20) ×10^9^], 3.50×10^9^/L [IQR: (2.70-4.80) ×10^9^], and 6.65×10^9^/L [IQR: (3.51-9.70) ×10^9^], respectively; the critical group showed significantly higher values than the mild and severe groups (P<0.001). The median lymphocyte levels in the mild, severe, and critical groups were 1.26×10^9^ /L [IQR: (0.90-1.60) ×10^9^], 0.98×10^9^/L [IQR: (0.70-1.26) ×10^9^], and 0.54×10^9^/L [IQR: (0.45-0.80) ×10^9^], respectively. The critical group showed significantly lower values than the mild and severe groups (P<0.001). The platelet levels were lower in the critically group than the mild and severe groups, but were still within the normal range. The levels of lactate dehydrogenase, creatinine, C-reactive protein, and procalcitonin increased with increasing illness severity (P<0.05). There were no significant differences in the blood test results across the three groups, including the values of albumin, alanine aminotransferase, aspartate aminotransferase, total bilirubin, potassium, sodium, and blood urea nitrogen. Multiple mottling and ground-glass opacity were typical imaging manifestations noted in patients with COVID-19, and their prevalence rates in the mild, severe, and critical groups were 24.29%, 42.86%, and 68.97%, respectively (*P*<0.001).

**Table 2:** Laboratory and radiograph findings of the different subtypes in older patients with COVID-19.

**Characteristic**	**Mild type (n=140)**	**Severe type (n=63)**	**Critical type (n=29)**	***P* value**
**Blood routine**				
Leucocyte count (×10^9^/L)	5.20(4.38-6.48)	5.0(4.1-6.88)	8.08(4.4-10.8)	0.02
Neutrophil count (×10^9^/L)	3.22(2.59-4.20)	3.50(2.70-4.80)	6.65(3.51-9.70)	<0.001
Lymphocyte count (×10^9^/L)	1.26(0.90-1.60)	0.98(0.70-1.26)	0.54(0.45-0.80)	<0.001
Neutrophil count/lymphocyte count	2.45(1.82-3.65)	4.08(2.39-6.20)	9.67(6.86-21.10)	<0.001
Hemoglobin (g/L)	125.0(113.0-138.0)	122.0(113.5-133.5)	121.0(110.5-137.5)	0.535
Platelet count (×10^9^/L)	204(170-279)	175(139-236)	156(123-191)	<0.001
**Coagulation function**				
International normalized ratio	1.02(0.96-1.06)	1.01(0.96-1.10)	1.0(0.97-1.06)	0.895
**Blood biochemistry**				
Albumin (g/L)	38.40(35.43-41.25)	36.30(33.30-39.50)	34.60(30.65-38.45)	0.001
Alanine aminotransferase (U/L)	25(16-36)	24(16-31)	21(14-31)	0.664
Aspartate aminotransferase (U/L)	25(20-33)	25(19-34)	29(18-38)	0.891
Total bilirubin (umol//L)	9.70(7.0-12.55)	10.10(7.90-13.15)	9.10(5.70-14.30)	0.671
Potassium (mmol/L)	3.99(3.70-4.37)	3.89(3.45-4.25)	3.81(3.50-4.14)	0.072
Sodium (mmol/L)	138.0(135.72-140.15)	137.50(134.95-140.0)	136.0(130.60-139.0)	0.027
Blood urea nitrogen (mmol/L)	4.51(3.83-5.47)	4.59(3.60-7.10)	6.16(4.48-8.72)	0.032
Creatinine (umol/L)	64.0(54.0-76.5)	68.0(57.0-84.0)	76.0(63.0-96.5)	0.003
Creatinine kinase (U/L)	56.50(41.25-88.75)	62.0(26.25-113.75)	80.0(52.0-173.50)	0.038
Lactate dehydrogenase (U/L)	218.0(175.0-256.50)	233.0(190.0-313.0)	273.0(243.0-354.0)	<0.001
**Infection-related biomarkers**				
C-reactive protein (mg/L)	16.02(4.41-39.26)	19.10(5.89-44.70)	41.86(6.33-70.10)	0.039
Procalcitonin (ng/mL)	0.09(0.04-0.14)	0.05(0.04-0.08)	0.19(0.04-0.25)	0.046
**Chest radiography/Computed tomography findings**				
Multiple mottling and ground-glass opacity	34(24.29)	27(42.86)	20(68.97)	<0.001

Data are presented as medians (interquartile ranges), n (%) and n/N (%).

### Treatment and outcomes

All patients were isolated in designated hospitals and received supportive care as well as the currently recommended medications. As shown in Table 3, 135 cases (84.77%), 60 cases (95.24%), and 29 cases (100%) received antiviral treatment, including interferon-α sprays, arbidol hydrochloride capsules, and lopinavir and ritonavir tablets in the mild, severe, and critical groups, respectively (*P*=0.504). The durations from illness onset to antiviral therapy initiation were 4 days (IQR: 2.0-7.0), 5 days (IQR: 1.5-8.5), and 4 days (IQR: 2.0-8.0) in the mild, severe, and critical groups, respectively (*P*=0.390). With increases in the illness severity, the proportion of the use of glucocorticoids and intravenous immunoglobins rose (*P*<0.001). Ten patients received extracorporeal membrane oxygenation (ECMO) therapy, and six underwent continuous renal-replacement therapy (CRRT) in the critical group; none of the patients received ECMO therapy and only one underwent CRRT in the severe group. Three patients had shock in the critical group, while there were no cases with shock in the mild and severe groups (*P*<0.001). The viral RNA shedding durations were 16 days (IQR: 12-22), 17 days (IQR: 14-21), and 25 days (IQR: 17-30) in the mild, severe, and critical groups, respectively (P<0.001).

**Table 3:** Treatments and clinical outcomes of the different subtypes in older patients with COVID-19.

**Characteristic**	**Mild type (n=140)**	**Severe type (n=63)**	**Critical type (n=29)**	***P* value**
Shock	0(0)	0(0)	3(10.34)	<0.001
Time from illness onset to antiviral treatment initiation (days)	4.0(2.0-7.0)	5.0(1.5-8.5)	4.0(2.0-8.0)	0.390
Antiviral treatment	135(96.43)	60(95.24)	29(100)	0.504
Viral RNA shedding time	16(12-22)	17(14-21)	25(17-30)	<0.001
Glucocorticoids	22(15.71)	29(46.03)	26(89.66)	<0.001
Use of intravenous immunoglobulin	17()	21()	23(79.31)	<0.001
Use of extracorporeal membrane oxygenation	0(0)	0(0)	10(34.48)	<0.001
Use of continuous renal-replacement therapy	0(0)	1(1.59)	6(20.69)	<0.001
Clinical outcomes at data cutoff				
Discharge from hospital	140(100)	63(100)	20(68.97)	0.098
Hospitalization	0(0)	0(0)	8(27.59)	0.098
Number of days in hospital	18(14-23)	22(19-26)	32(21-68)	<0.001
Lung transplantation	0(0)	0(0)	2(6.90)	0.001
Death	0(0)	0(0)	1(3.45)	0.030

Data are presented as medians (interquartile ranges), n (%) and n/N (%).

By April 13, one patient had died, two had received lung transplantation, and eight remained hospitalized in the critical group. By May 27, among the eight patients who were still hospitalized, two withdrew from the ECMO treatment and were transferred to the general ward, while the other six patients were still receiving the ECMO therapy. In the other two groups, all patients had survived and were discharged. The number of days of hospitalization were 18 days [IQR: 14-23], 22 days [IQR: 19-26], and 32 days [IQR: 21-68] in the mild, severe, and critical groups, respectively (P<0.001).

### Risk factors associated with progression to critical illness

Univariate Cox regression was used to analyze the risk factors for critical illness in the older patients with COVID-19, as shown in Table 4. Older age was shown to increase the likelihood of critical illness even in older patients (≥60 years) (hazard ratio [HR] 1.107, confidence interval [CI] 1.065-1.151, *P*<0.001). Shortness of breath as a symptom (HR 11.328, CI 5.370-23.894, P<0.001), and comorbidities including hypertension (HR 3.563, CI 1.578-8.047, *P*=0.002), heart disease (HR 9.638, CI 4.626-20.081, *P*<0.001), and COPD (HR 7.108, CI 2.891-17.481, *P*<0.001) were predictive of critical illness. The increasing odds of critical illness development in patients with COVID-19 were associated with higher NLR values (HR 1.157, CI 1.117-1.199, *P*<0.001), lower albumin levels (HR 0.875, CI 0.807-0.950, *P*<0.001), higher C-reactive protein levels (HR 1.012, CI 1.005-1.020, *P*=0.002), and multiple mottling and ground-glass opacity (HR 4.573, CI 2.082-10.045, P<0.001). In the multivariate analysis, only older age (HR 1.121, CI 1.070-1.174, *P*<0.001), heart disease (HR 2.587, CI 1.156-5.787, *P*=0.021), higher NLRs (HR 1.136, CI 1.094-1.180, *P* < 0.001), and multiple mottling and ground-glass opacity (HR 4.518, CI 1.906-10.712, P<0.001) remained predictors of critical illness when the other factors in the model were kept constant.

**Table 4:** Risk factors for critical illness.

**Variables**	**Mild/Severe type (n=203)**	**Critical type (n=29)**	**Univariate analysis**		**Multivariate analysis**	
			**HR (95% CI)**	***P*-value**	**HR (95% CI)**	***P*-value**
Age (years)	66(63-70)	72(68-81)	1.107(1.065-1.151)	<0.001	1.121(1.070-1.174)	<0.001
Time from illness onset to first hospital admission (days)	3(1-7)	3(1-5)	0.937(0.836-1.049)	0.258		
Hypertension	79(38.92)	21(72.41)	3.563(1.578-8.047)	0.002		
Heart disease	15(7.39)	16(55.17)	9.638(4.626-20.081)	<0.001	2.587(1.156-5.787)	0.021
COPD	3(1.48)	6(20.69)	7.108(2.891-17.481)	<0.001		
Shortness of breath	8(3.94)	12(41.38)	11.328(5.370-23.894)	<0.001		
NLR	2.68(1.96-4.42)	9.67(6.86-21.10)	1.157(1.117-1.199)	<0.001	1.136(1.094-1.180)	<0.001
Albumin (g/L)	38.0(35.20-41.0)	34.60(30.65-38.45)	0.875(0.807-0.950)	0.001		
C-reactive protein (mg/L)	16.95(4.75-40.62)	41.86(6.33-70.10)	1.012(1.005-1.020)	0.002		
Multiple mottling and ground-glass opacity	61(30.05)	20(68.97)	4.573(2.082-10.045)	<0.001	4.518 (1.906-10.712)	0.001

HR, hazard ratio; CI, confidence interval; COPD, chronic obstructive pulmonary disease; NLR, neutrophil-to-lymphocyte ratio.

### Association of the NLR with progression to critical illness

**Figure 1:**
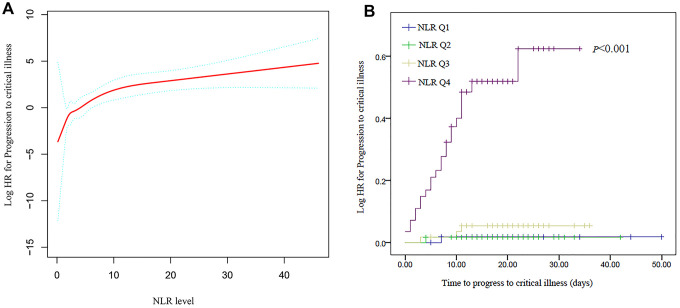
**Association between the neutrophil-to-lymphocyte ratio (NLR) and progression to critical illness.** (**A**) Adjusted hazard ratio (HR) for progression to critical illness according to the NLR. (**B**) Cumulative probability of progression to critical illness with increasing NLR values.

Figure 1A shows the association between the NLR and progression to critical illness, as identified using a Cox proportional hazards model adjusted for the baseline covariates. For the sensitivity analysis, we converted the NLR from a continuous variable to a categorical variable (the quartile of NLR), and the P for trend of the NLR with categorical variables in the fully adjusted model (model II) was consistent with that obtained when the NLR was a continuous variable. The relationship between the NLR and progression was significant and graded (HR: 1.16 per unit; 95% CI: 1.10-1.22; P<0.001). When adjusted for sex and age, the ratio of the highest quartile of the NLR compared to the lowest quartile was 33.017 (95% CI 4.436-245.732, P <0.001), and in the fully adjusted model, the odds of the NLR as a clinical risk factor was 21.755 (95% CI 2.854-165.860, P<0.001) (Table 5). Figure 1B shows the Kaplan-Meier analyses graphs for progression to critical illness based on the quartiles of the NLR.

**Table 5:** Relationships between the neutrophil-to-lymphocyte ratio and critical disease development using different models.

**Neutrophil-to-lymphocyte ratio (quartile)**	**Total, n**	**Event (%)**	**HR (95% CI)**		
			**Crude Model**	**Model I**	**Model II**
Q1	58	1(1.72)	Reference	Reference	Reference
Q2	60	1(1.61)	0.980(0.061-15.662)	1.186(0.074-18.984)	1.324(0.081-21.591)
Q3	57	3(5.26)	2.914(0.303-28.014)	2.966(0.308-28.533)	3.867(0.399-37.461)
Q4	57	24(42.11)	29.769(4.024-220.233)	33.017(4.436-245.732)	21.755(2.854-165.860)
*P* for trend	—	—	<0.001	<0.001	<0.001
Increase per unit	—	—	1.16(1.12-1.20)	1.15(1.11-1.19)	1.16(1.10-1.22)

Note: Model I adjusted for age, sex.

Model II adjusted for age, sex, hypertension, heart disease, COPD, shortness of breath, albumin, C-reactive protein and multiple mottling and ground-glass opacity.

HR, hazard ratio; CI, confidence interval; COPD, chronic obstructive pulmonary disease.

## DISCUSSION

In the present study, we described the clinical characteristics and outcomes of older patients who had COVID-19 with the highest risk of critical illness after SARS-CoV-2 infection. Of the 232 older patients with COVID-19, 29 (12.5%) had critical disease; one patient died and two received lung transplantation in the critical group. Eight patients remained in the hospital, and received ECMO therapy for more than two weeks. The median duration of hospitalization was 32 days in the critical group, which was significantly longer than that in the mild and severe groups.

Disease typing and prognostic indicators are of great significance in the guidance of classified treatment and prevention of medical runs, and saving patients with a critical status. In our study, some independent risk factors for progression to critical illness were found using multivariate Cox regression analysis, such as older age, multiple mottling and ground-glass opacity, heart disease, and a high NLR.

Previously, older age was reported as an important independent predictor of fatal outcomes in patients with COVID-19 [18–21]. Older age was shown to increase the likelihood of critical illness even in older patients (HR 1.107, CI 1.065-1.151, P<0.001). Our results are consistent with those of previous reports [13]. Elderly patients experience a marked cell-mediated immune function decline and a reduced degree of humoral immune function. The cytokine and chemokine signaling networks are altered in elderly patients, and tend to favor a type 2 cytokine response over type 1 cytokine responses, potentially leading to poor outcomes [22].

Advanced imaging in patients with COVID-19 is capable of demonstrating disease progression. Generally, imaging manifestations are in line with the severity of COVID-19 [23]. Zhong et al. found that the computed tomography (CT) images in patients with different clinical types of COVID-19 had characteristic manifestations, and that the presence of solid shadows may be predictive of severe and critical illness [24]. Our study found that the presence of multiple mottling and ground-glass opacity on CT was an independent predictor of progression to critical illness (HR 4.518, CI 1.906-10.712, *P*=0.001). We also found that older patients with COVID-19 who had heart disease were likelier to progress to critical illness. Several studies have shown that coexisting heart disease was an independent risk factor associated with fatal outcomes in patients with COVID-19 [12, 25]. Cardiac complications, including new or worsening heart failure, new or worsening arrhythmia, and myocardial infarction are commonly observed in patients with severe pneumonia. Cardiac arrest occurs in about 3% of inpatients with severe pneumonia [26].

Chen et al. showed that, compared to cases with moderate disease severity, those with a severe disease status more frequently had lymphopenia [27]. Mo et al. found that patients with refractory disease had higher neutrophil levels than general COVID-19 patients [28]. The prognostic role of the NLR has been documented in multiple settings, including malignancies, infectious diseases, liver cirrhosis, and cerebrovascular disease [29–32]. In this study, we investigated the correlation of the NLR with critical illness in older patients with COVID-19 to evaluate the prognostic power of the NLR at admission in the prediction of progression to critical illness. In the sensitivity analysis, we converted the NLR from a continuous variable to a categorical variable, and found that the higher the NLR the greater the likelihood of progression to critical illness. Liu et al. also found that the NLR is an independent risk factor of in-hospital mortality in COVID-19 patients, especially male patients [17]. Our previous study suggested that a change in the NLR on admission among older patients with COVID-19 might be a biomarker specific to the prediction of progression to critical illness. A future study, conducted to elucidate this specificity, will further our understanding of the prognostic value of the NLR.

Our study has several limitations. First, its retrospective nature may decrease the accuracy of the findings; there is a need for a validation cohort to assess the predictive accuracy and confirm our findings. Second, owing to the retrospective design, data on some relevant factors such as interleukin-6 and D-dimer were incomplete and could not be included in the risk factor analysis. Third, data on the outcomes of older patients with COVID-19 in the critical group require further investigation, as, at the time of this study, there were still eight patients who were undergoing treatment at the hospital.

## MATERIALS AND METHODS

### Patients

This retrospective study, focusing on the epidemiological and clinical characteristics of older (age≥60 years) patients with confirmed COVID-19, was conducted from January 17 to March 3, 2020. All the enrolled cases showed real-time reverse transcriptase polymerase chain reaction (RT-PCR) positivity for SARS-CoV-2, and were retested several times during their hospitalization. Data were collected uniformly by the Health Commission of Zhejiang Province, wherein all patients were assigned to specific hospitals for unified treatment according to Zhejiang Province’s emergency rule. The diagnosis of COVID-19 infection was based on the interim guidance of the World Health Organization (WHO) [33], and all data were shared with the WHO, with the primary analytic results reported to the authority of Zhejiang Province. Since the collection and analysis of all cases were determined by the Health Commission of Zhejiang Province under national authorization and considered as part of the continuing public health outbreak investigation, our retrospective study was exempt from institutional review board approval.

The subtype definition of COVID-19 patients was based on the diagnosis and treatment scheme for COVID-19 in China, based on a minor modification of WHO standards [34]. The degree of COVID-19 was categorized as mild, severe, or critical: the mild type included non-pneumonia and mild pneumonia cases, and the severe type was characterized by dyspnea, respiratory frequency ≥30 min, blood oxygen saturation ≤93%, PaO2/FiO2 ratio <300, and/or rate of lung infiltration >50% within 24–48 h. Critical cases were those that exhibited respiratory failure, septic shock, and/or multiple organ dysfunction/failure.

### Procedures

We obtained epidemiological, demographic, laboratory, clinical, management, and outcome data from patients’ medical records. Data were retrieved and reviewed by two independent observers. Clinical outcomes were followed-up until April 13, 2020. Missing or unclear data were confirmed by direct communication with healthcare providers. Throat swab specimens obtained from the upper respiratory tract and sputum of all patients were collected at admission. Laboratory confirmation of COVID-19 was performed at the First Affiliated Hospital at Zhejiang University, under the authorization of the Centers for Disease Control and Prevention at the Zhejiang Province/city level, by previously reported RT-PCR methods. All patients underwent chest CT at admission. Patients with other common respiratory viruses, including respiratory syncytial virus, parainfluenza virus, influenza A and B virus, and adenovirus were excluded from this study.

### Data collection

In this study, we collected data on epidemiology, anthropometrics, demographics, as well as symptoms and signs at the time of admission to the hospital. We analyzed the blood collected within 48 hours of admission. Additional data collected included those on the results of laboratory tests and chest CT, comorbidities, co-infection with other respiratory pathogens, treatment (including drugs, intensive care and mechanical ventilation), and other clinical outcomes.

### Statistical analysis

Continuous variables are expressed as medians (range), and were compared using t tests or Mann-Whitney U tests, and categorical variables were compared using chi-squared tests or Fisher’s exact tests. Follow-up was initiated on the day of admission, and ended at the patient’s death or until the last follow-up. The Kaplan-Meier method was used to evaluate the cumulative rate of progression to critical illness, and a log-rank test was used to assess differences between groups. HRs were calculated using the Cox regression model. Variables with *P* < 0.05 in the univariate analysis were included in a stepwise Cox proportional hazards regression model. We performed tests for linear trend by entering the median value of each quartile of the NLR as a continuous variable in the models. The Cox proportional hazards model was used to estimate the HRs associated with the NLR for the risk of progression to critical illness with adjustment for pertinent variables. The HRs and 95% CIs of the progression to critical illness in each subgroup were estimated, and their interactions tested. Statistical analyses were conducted using SPSS version 26.0 (IBM Corporation, Armonk) and R version 3.4 (R Foundation). A two-sided *P* value < 0.05 was considered to indicate statistical significance.
